# *Salmonella* Kingabwa Infections and Lizard Contact, United States, 2005

**DOI:** 10.3201/eid1304.060888

**Published:** 2007-04

**Authors:** Sharon Greene, Anthony Yartel, Kerry Moriarty, Laura Nathan, Ellen Salehi, Leslie Tengelsen, Nehal Patel, Michael Lynch

**Affiliations:** * Centers for Disease Control and Prevention, Atlanta, Georgia, USA; † Maine Center for Disease Control and Prevention, Augusta, Maine, USA; ‡ County of San Diego Health and Human Services Agency, San Diego, California, USA; § Arizona Department of Health Services, Phoenix, Arizona, USA; ¶ Ohio Department of Health, Columbus, Ohio, USA; # Idaho Department of Health and Welfare, Boise, Idaho, USA

**Keywords:** Lizards, *Salmonella* Infections, Child, letter

**To the Editor:** Nontyphoidal *Salmonella* infections cause an estimated 1.4 million illnesses and 400 deaths in the United States annually (*1*). Among the >2,500 *Salmonella* serotypes, *Salmonella enterica* serotype Kingabwa rarely causes human illness. This serotype was first reported in a patient in the Belgian Congo in 1953 (*2*). From 1995 through 2004, only 30 human illnesses caused by *S.* Kingabwa were reported to the National *Salmonella* Surveillance System (*3*). No common source for *S.* Kingabwa human illnesses has been previously identified. We recently investigated an outbreak of *S.* Kingabwa infections associated with 2 lizard species: the water dragon and the bearded dragon.

Eighteen isolates of *S.* Kingabwa (antigenic formula: I 43:y:1,5) were received by PulseNet, the National Molecular Subtyping Network for Foodborne Disease Surveillance, from 2001 through 2005. When digested with restriction enzyme *Xba*I and subtyped by pulsed-field gel electrophoreisis (PFGE), 13 isolates produced a single, indistinguishable pattern (KINX01.0001). Of these, 1 (8%) was isolated in 2001, 4 (31%) were isolated in 2002, 2 (15%) were isolated in 2004, and 6 (46%) were isolated in 2005. We defined a case as illness during 2005 caused by *S.* Kingabwa that matched pattern KINX01.0001 by PFGE. Of the 9 *S.* Kingabwa isolates received by PulseNet in 2005, 6 matched KINX01.0001. Antimicrobial drug susceptibility of 3 isolates was determined by the National Antimicrobial Resistance Monitoring System (NARMS) for Enteric Bacteria at the Centers for Disease Control and Prevention (CDC), and the isolates were susceptible to each of 15 antimicrobial agents tested.

The 6 patients in the 2005 outbreak did not know each other and resided in 5 states: Maine (2 patients), Arizona, California, Idaho, and Ohio. Illness onset dates were in June, July, August, October (2 patients), and November 2005. Of the 6 patients, 4 (67%) were ≤1 year old (range <1–53 years), 4 were male, 2 were hospitalized, and none died.

Interviews with patients or their parents or guardians conducted during routine public health surveillance collected information on specific food items, water sources, restaurant venues, travel history, and animal contact. No common food or environmental source was identified. However, 4 (67%) of the 6 patients had known exposure to lizards: 3 water dragons (*Physignathus cocincinus*, Figure) and 1 bearded dragon (*Pogona* sp.). Of these 4 patients, 3 had >1 lizard in their own household as pets; the other patient was exposed to a lizard when visiting a family member. The 2 patients who did not recall lizard exposure might represent patients with background cases unrelated to lizards. Single cultures of the 2 lizards available for testing in February 2006 did not yield *S.* Kingabwa, which could mean that they did not carry this rare *Salmonella* serotype. However, this does not exclude lizards as the source of these illnesses because lizards intermittently shed salmonellae (*4*).

**Figure:**
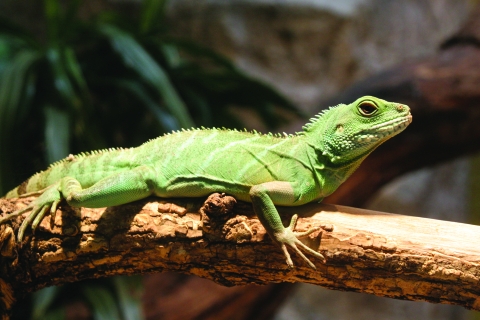
Water dragon (*Physignathus cocincinus*). Three of the patients with *Salmonella* Kingabwa infections were exposed to this reptile species. Photo credit: Robert Lawton, rklawton@LawtonPhotos.com.

The lizards had been purchased from local pet shops and a traveling reptile show. Shipments of reptiles were mixed together at points of sale, and numerous distributors and importers were used, so determining the origin of individual reptiles was not feasible. However, water dragons and bearded dragons are imported into the United States from Asia and Australia and are rarely bred domestically.

Two thirds of the patients in this outbreak had documented exposure to 1 of 2 lizard species, and half of the patients had pet lizards in their homes. In 2001, the estimated number of households with lizards was 545,000, which represents ≈0.5% of all American households (*5*). Using a standard binomial model, the probability of finding at least 3 of 6 persons chosen at random to be lizard owners is 0.000002. To our knowledge, this is the first investigation to identify a strong association between the rare serotype *S.* Kingabwa and lizards and the first instance of which we are aware that a serotype has been associated with a particular species of lizard dispersed in homes across the United States.

The association between reptile exposure and human salmonellosis is well-established (*6*–*8*). CDC has published recommendations for reducing the risk for infection from reptiles (http://www.cdc.gov/healthypets/animals/reptiles.htm); these include thoroughly washing hands with soap and water after handling reptiles or their cages and keeping reptiles out of food preparation areas. The young age of most patients in this outbreak supports the recommendation that reptiles should not be allowed in households with children <5 years of age.
